# Standard rectal swabs as a surrogate sample for gut microbiome monitoring in intensive care

**DOI:** 10.1186/s12866-022-02487-0

**Published:** 2022-04-12

**Authors:** Sanmarié Schlebusch, Rikki M. A. Graham, Amy V. Jennison, Melissa M. Lassig-Smith, Patrick N. A. Harris, Jeffrey Lipman, Páraic Ó Cuív, David L. Paterson

**Affiliations:** 1 University of Queensland Centre for Clinical Research, Herston, Brisbane, Queensland Australia; 2 Q-PHIRE Genomics and Public Health Microbiology, Forensic and Scientific Services, Coopers Plains, Brisbane, Queensland Australia; 3 Pathology Queensland, Queensland Health, Herston, Brisbane, Queensland Australia; 4 Intensive Care Services, Royal Brisbane and Women’s Hospital, Brisbane, Queensland Australia; 5 Jamieson Trauma Institute, Royal Brisbane and Women’s Hospital, Brisbane, Queensland Australia; 6 Nîmes University Hospital, University of Montpellier, Nîmes, France; 7 Mater Research Institute, Translational Research Institute, University of Queensland, Brisbane, Queensland Australia

**Keywords:** Gut microbiome, Antibiotic effect, Rectal swab, ICU

## Abstract

**Background:**

The purpose of this study was to investigate the use of routinely available rectal swabs as a surrogate sample type for testing the gut microbiome and monitoring antibiotic effects on key gut microorganisms, of patients hospitalised in an intensive care unit. A metagenomic whole genome sequencing approach was undertaken to determine the diversity of organisms as well as resistance genes and to compare findings between the two sampling techniques.

**Results:**

No significant difference was observed in overall diversity between the faeces and rectal swabs and sampling technique was not demonstrated to predict microbial community variation. More human DNA was present in the swabs and some differences were observed only for a select few anaerobes and bacteria also associated with skin and/or the female genitourinary system, possibly reflecting sampling site or technique. Antibiotics and collections at different times of admission were both considered significant influences on microbial community composition alteration. Detection of antibiotic resistance genes between rectal swabs and faeces were overall not significantly different, although some variations were detected with a potential association with the number of human sequence reads in a sample.

**Conclusion:**

Testing the gut microbiome using standard rectal swab collection techniques currently used for multi-resistant organism screening has been demonstrated to have utility in gut microbiome monitoring in intensive care. The use of information from this article, in terms of methodology as well as near equivalence demonstrated between rectal swabs and faeces will be able to support and potentially facilitate the introduction into clinical practice.

**Supplementary Information:**

The online version contains supplementary material available at 10.1186/s12866-022-02487-0.

## Background

There has been great interest in the gut microbiome, and its role in human health and disease has been well described [1]. Disruption of the gut microbiome due to antibiotics increases the risk of infection and evolution of multi-drug resistant (MDR) bacteria [2]. MDR bacteria are a known consequence of antimicrobial therapy and common in healthcare-associated infections especially in critically ill patients [2]. The use of gut microbiome testing to assess the effects of antibiotics and assist in antibiotic selection has shown promise but is not yet widely in use [2].

One of the challenges of faeces collection for microbiome testing during hospitalisation is that bowel motions of patients are often infrequent, particularly in critically ill patients where up to 70% of patients may be constipated, hence unable to provide stool for analysis [3, 4]. Rectal swabs may be an alternative solution for microbiome testing for such patients [5, 6]. However, conflicting reports of reliable use of rectal swabs have been reported [7, 8]. Fair *et al* [9] for example described some limitations in the use of rectal swabs from critically ill patients using 16S rRNA targeted sequencing, although rectal swabs and stool samples were unable to be collected at the same time.

Although swabs with specific transport media have been described for gut microbiome testing [6], these are not widely in use or routinely available, and can therefore introduce an additional process or cost to healthcare practice. This study assessed whether the standard dry cotton swabs already used in hospitals for microbiological testing would be suitable as alternative to faeces collection for assessment of the effects of antibiotics on the gut microbiome.

This study has collected simultaneous faeces and rectal swabs from hospitalised patients to assess the effects of sampling technique on microbial diversity and antibiotic effect observations on the gut microbiome using a metagenomic approach and to establish methodology for ongoing use in practice. The aim was to observe whether the impact of antibiotic treatments on the gut microbiome could be assessed with rectal swabs and findings would be comparable to those determined with faeces sampling.

Firstly the study was used to observe microbial diversity of samples collected with the two techniques and whether microbial diversity alterations due to antibiotic effect or at different time points for patients with multiple collections were affected by sampling techniques. Secondary objectives included assessing and describing the changes to the relative abundance in the metagenome of key organisms associated with antibiotic effect, Enterobacterales, *Clostridioides difficile*, *Pseudomonas* spp., *Acinetobacter* spp., *Candida* spp., and organisms considered to be anaerobic (Bacteroidales, Bifidobacteriales, Clostridiales and Fusobacteriales); as well as detectable Gram-negative resistance genes in the gut metagenome, including extended spectrum β-lactamase (ESBL) and carbapenemase genes [10, 11].

While most other studies assessed the utility of rectal swabs for gut microbiome testing with 16S rRNA gene-based sequencing or only focused on microbial community composition, we used whole genome sequencing metagenomics (WGSM) to determine not only organism composition but also to compare the detection of resistance genes using faeces and rectal swabs, which will inform the use of rectal swab WGSM for studying antibiotic effect on the gut microbiome in ICU patients.

## Results

A total of 36 samples, comprising 18 faeces and 18 rectal swabs collected within about 24 hours of each other, from 11 patients (7 males and 4 females) were included in this study analysis. The average age for patients was 57 years (median 56), ranging from 28 to 88 years.

**Fig. 1:**
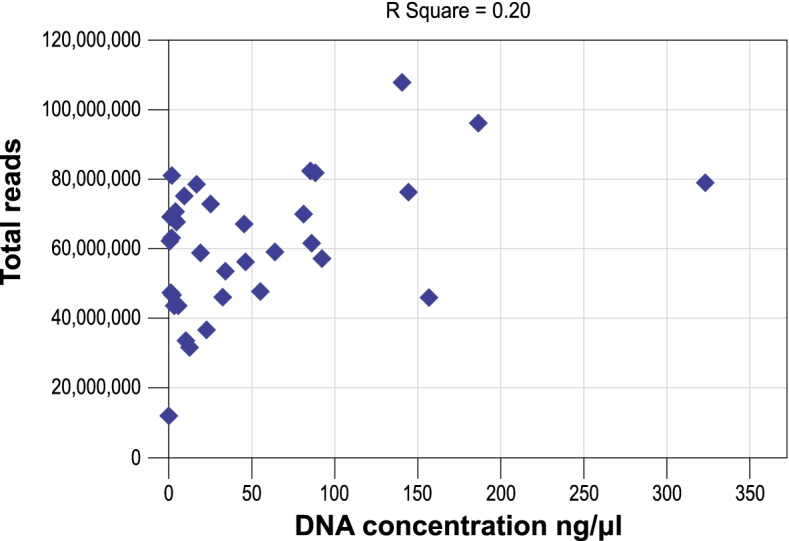
Scatterplot demonstrating the relationship between DNA concentration (ng/μL)(*x-*axis) and total number of reads for each sample (*y-*axis). R Square = 0.20.

The average DNA concentration from faeces was 86.61 ng/μL and from swabs 13.77 ng/μL, however DNA concentration did not correlate directly with total number of sequencing reads achieved (Fig. 1). Average DNA concentration from swabs was 16% of average faeces DNA concentration, but sequence reads achieved were 90% (57,616,320 reads from swabs compared to 64,170,721 for faeces).

Total number of reads ranged from 31,542,724 to 107,805,306 for faeces and 12,022,910 to 82,306,018 for rectal swabs (Table 1), noting that libraries were normalised before loading. A higher percentage sequences mapped to the human genome in the rectal swabs, 23.41% (1.78 – 92.38%), compared to faeces, 5.54% (1.60 – 15.87%)( t-Test *p*-value = 0.03).

**Table 1:** Average DNA concentration (ng/μl), read numbers and diversity for faeces and rectal swab groups

	DNA concentration		Total reads		Unmapped reads^a^		Shannon index	
	Faeces	Swabs	Faeces	Swabs	Faeces	Swabs	Faeces	Swabs
Average	86.61	13.77	64,170,721	57,616,320	60,787,262	44,442,521	3.20	3.44
Min	2.07	0.03	31,542,724	12,022,910	28,450,859	6,171,540	0.77	1.13
Max	323.20	85.20	107,805,306	82,306,018	103,450,092	77,005,487	4.27	4.28

^a^Unmapped reads refers to reads not mapped to the reference human genome

**Fig. 2:**
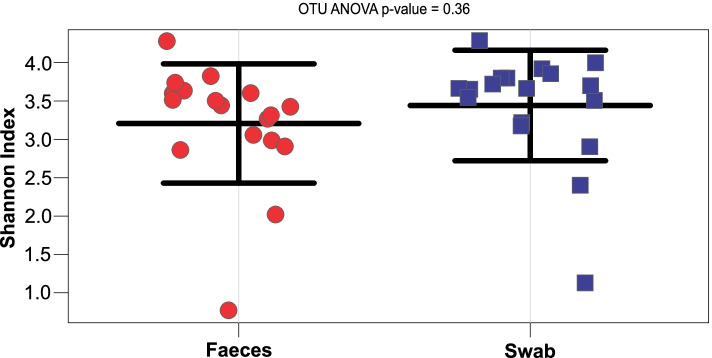
Stripchart demonstrating Shannon diversity index (*y-*axis) for faeces samples on the left (circles) (*n* = 18, average = 3.20) compared to swabs on the right (squares) (*n* = 18, average = 3.44). ANOVA *p* = 0.36.

**Fig. 3:**
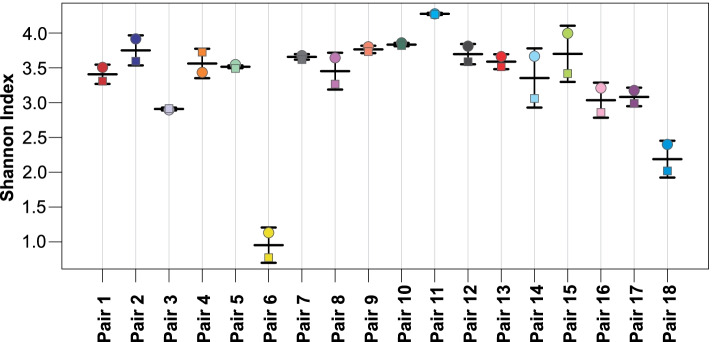
Stripchart demonstrating the Shannon diversity index (*y*-axis) for each faeces (square) and swab (circle) pair.

Microbial diversity was determined, and assesses the number as well as abundance distribution of organisms [12]. No statistically significant difference in OTU (operational taxonomic unit) diversity was observed for the determined Shannon index between faeces and rectal swab groups (ANOVA *P* = 0.36) (Fig. 2), with average values of 3.20 and 3.44 respectively (Table 1). Within each pair the Shannon index difference ranged from 0.01 to 0.61 (Fig. 3).

Pair 6 was observed to have the lowest diversity index, with 0.77 for the faeces and 1.13 for the swab. This was the second sample pair collected from a patient. Sample pair 5 was collected from the same patient two days after commencing piperacillin-tazobactam and sample pair 6 the day after completion of therapy, a further 5 days on piperacillin-tazobactam. The Shannon index was slightly higher in the swabs compared to the faeces for both pairs, differing by 0.05 and 0.36, respectively. The Shannon index dropped by 2.72 from the first to the second faeces, and by 2.42 from the first to the second rectal swab.

Other measures such as Chao1 and Richness confirmed the absence of any statistically significant differences in diversity between the faeces and rectal swab with *p*-values of 0.56 and 0.32 respectively. Seven patients had more than one pair of samples collected, with no statistically significant difference in the diversity in either the primary or the follow-up sample groups between the faeces samples and the rectal swabs (Shannon index *p* = 0.36 and *p* = 0.62 respectively).

**Fig. 4:**
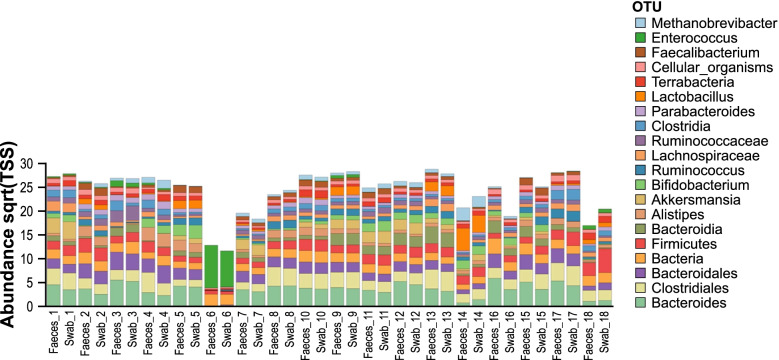
Clustered barchart quantitative visualisation of the composition of the top 20 OTU abundances (square root transformed (sqrt) and total sum scaling (TSS) normalised) for each sample. Samples are presented in their respective pairs. A colour legend indicates the taxonomic identification achieved for the presented OTUs to their lowest taxonomic classification respectively.

A Wilcoxon-rank test demonstrated 14 OTUs with abundance difference of *P* < 0.05 between the faeces and swab groups (Table 2), however when compensation with Bonferroni correction and FDR is applied, no OTUs with *p*-values of < 0.05 were observed. All of the OTUs listed in Table 3 were within the top 500 most abundant organisms and the maximum inclusive filter setting was used to ensure any potentially relevant differences were detected. The composition and abundance within pairs were further compared. The abundance for the top 20 most abundant taxa in each sample and grouped by pairs are demonstrated in Fig. 4.

**Table 2:** Wilcoxon-rank test faeces compared to swab abundance at maximum setting of top 10,000 most abundant organism OTUs, with *p* < 0.05

OTU	P (rank test)	Adjusted P (Bonferroni)	False discovery rate (FDR)	Faeces mean	Swab mean	Fold Change
*Finegoldia magna*	0.0002	0.08	0.08	0.01	0.16	-21.21
*Peptoniphilus*	0.0008	0.40	0.15	0.02	0.15	-8.15
*Lawsonella clevelandensis*	0.0009	0.46	0.15	0.03	0.16	-4.71
*Peptoniphilus grossensis*	0.002	1	0.27	0.002	0.11	-48.73
*Porphyromonas bennonis*	0.004	1	0.45	0.01	0.12	-9.77
*Levyella massiliensis*	0.006	1	0.50	0.01	0.17	-22.00
*Prevotella corporis*	0.009	1	0.69	0.01	0.15	-29.33
*Porphyromonas* sp.	0.02	1	0.80	0.01	0.15	-14.00
*Mobiluncus curtisii*	0.02	1	0.80	0.01	0.11	-19.90
*Ileibacterium massiliense*	0.02	1	0.80	0.004	0.09	-24.28
*Prevotella corporis*	0.02	1	0.90	0.002	0.10	-57.63
*Varibaculum cambriense*	0.02	1	0.92	0.01	0.14	-9.73
*Finegoldia magna*	0.03	1	0.92	0.01	0.16	-19.80
Actinobacteria unclassified	0.04	1	1	0.10	0.15	-1.47

Footnote: The presence of an organism name more than once reflects two different OTU sequences in the database

**Table 3:** Differences in relative abundance for key OTUs between faeces and swabs

	Enterobacterales		*Pseudomonas* spp		*C. difficile*		Bifidobacteriales		Bacteroidales		Clostridiales		*C. albicans*	
	Faeces	Swabs	Faeces	Swabs	Faeces	Swabs	Faeces	Swabs	Faeces	Swabs	Faeces	Swabs	Faeces	Swabs
Average	1.42	1.39	0.25	0.20	0.09	0.12	1.08	1.49	5.42	4.91	4.54	4.75	0.46	0.37
Min	0.04	0.14	0.02	0.02	0	0	0.02	0.02	0.04	0.16	0.13	0.27	0	0
Max	5.15	5.34	1.49	0.84	0.40	0.53	2.91	4.95	7.47	6.76	5.94	6.49	7.53	5.98
P (rank test)	0.87		0.73		0.26		0.48		0.07		0.65		0.77	
p (ANOVA)	0.96		0.64		0.46		0.29		0.42		0.68		0.86

The relative abundance in each sample for the key organisms specified in the study objectives were compared for each sample pair and between groups (Table 3, Supplementary Table 1). *Acinetobacter* spp. relative abundance was not sufficient across all samples for statistical analysis (below the filtering threshold). The following orders were included for interrogation of anaerobes: Bifidobacteriales, Bacteroidales, and Clostridiales. Fusobacteriales only included a single OTU at a very low average relative abundance <0.04 and it was therefore not included in analysis. The only *Candida* sp. present in the abundance calculations was *Candida albicans*. There was no statistically significant difference in abundance between faeces and swabs for Enterobacterales, *Pseudomonas, C. difficile*, Bifidobacteriales, Bacteroidales, Clostridiales, or *C. albicans* at their respective taxonomic levels (Table 3)*.*

Within each pair the abundance difference for each of these key taxonomic groups was further analysed (Table 4). The highest maximum relative abundance difference within a pair was for Bifidobacteriales, 3.92, highest in the swab. The second highest in-pair difference was in the same sample pair, for Bacteroidales, 2.81 with the highest abundance in faeces. *Gardnerella vaginalis was the* OTU with the highest abundance difference for this sample pair*.* This sample also had the highest number of reads mapped to the human genome and the highest time difference between faeces and rectal swab collections (24.5 hours). Interestingly, the second highest in-pair difference for the same taxonomic orders was in samples collected 7 days earlier from the same patient. Unclassified *Bacteroides* (OTU) had the highest abundance difference in this earlier sample pair, and the notable human reads disproportion was not observed (Supplementary Table 1). This female patient was not on any antibiotics. There was no significance revealed between the swabs and faeces from the combined collections from this patient for Bacteroidales or Bifidobacteriales (Rank test *p* = 0.33 for both orders). *G. vaginalis* was present in both faeces and rectal swab samples, but with a dominant abundance in the rectal swab from the second paired collection. The most abundant species in the order Bacteroidales in both samples belonged to the genus *Bacteroides.* The most abundant species within the order Bifidobacteriales were *Bifidobacterium* spp. and *G. vaginalis.* This was the only patient with *G. vaginalis* with a relative abundance >0.01 in this study. The third highest maximum absolute difference within a pair was observed for the Clostridiales, 1.77, higher in faeces, and was in a patient on 6-hourly piperacillin-tazobactam with the swab collected 9.5 hours after the faeces. The highest impact of in-pair differences was observed for *Pseudomonas* spp. and Bifidobacteriales with the Pearson Correlation the lowest for these OTUs, 0.58 and 0.73 respectively. The maximum in-pair difference for Bifidobacteriales was for the sample pair described above and for the *Pseudomonas* spp. was from a patient that had the faeces sample collected 2 hours after commencement of meropenem and the swab 8 hours after the faeces and following a second dose of meropenem. There was however no statistically significant difference between the faeces and rectal swab groups overall for these OTUs (Table 3).

**Table 4:** In-pair differences for relative abundance per key organism OTU group

	Enterobacterales	*Pseudomonas*	*C. difficile*	Bifidobacteriales	Bacteroidales	Clostridiales	*C. albicans*
Average	0.26	0.14	0.04	0.45	0.74	0.45	0.09
Min	0	0	0	0	0.04	0.05	0
Max	1.39	1.32	0.17	3.92	2.81	1.77	1.55

To exclude the potential impact of antibiotic treatment on observations of faeces and swab comparison, the five pairs of samples from 3 patients that did not receive antibiotics were further analysed. The average absolute in pair differences for Enterobacterales, *C. difficile, Pseudomonas* spp., Bifidobacteriales, Bacteroidales, and Clostridiales were 0.08, 0.02, 0.05, 1.11, 1.16, and 0.39, respectively. *C. albicans* was only present in one pair with no difference in abundance.

Therefore, the in-pair difference noted for *Pseudomonas* in the overall group was likely due to antibiotic effect. No significant difference in diversity between the faeces and rectal swabs was demonstrated in this sub-cohort (Shannon index *p* = 0.43).

Eight pairs (from 5 patients) had faeces and swabs collected at the same time. Of these, five pairs were collected during or after antibiotics. The remaining ten pairs (7 patients) were collected at different times ranging from 10 minutes to 24 hrs 30 minutes. Of these 8 pairs were collected during or after antibiotics. Of the 10 pairs collected at different times, 7 faeces were collected before the swabs and 3 swabs before the faeces. There was no significant variation in the diversity differences between the swabs and faeces between these two groups (Shannon index ANOVA *p* = 0.97). There was no significant difference in diversity between the faeces and rectal swabs collected at the same time (Shannon index ANOVA *p* = 0.82) or at different times (Shannon index ANOVA *p* = 0.15).

**Fig. 5:**
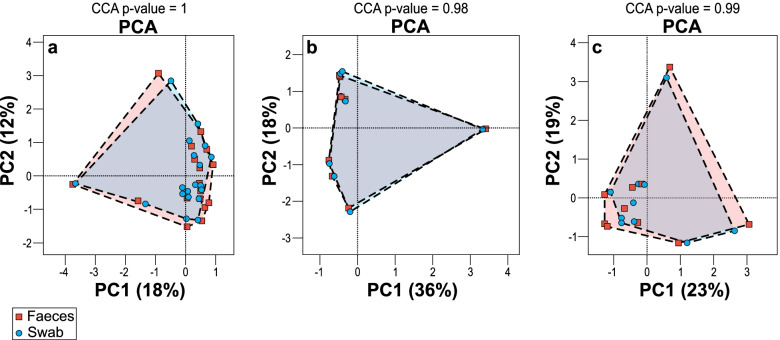
Principal Component Analysis (PCA) of OTU abundance for faeces (squares) compared to swabs (circles). **a** Overall comparison of all samples (*n *= 36) for each collection type. Canonical correspondence analysis (CCA) *p* = 1, **b** comparison of samples collected at the same time (*n *= 16)(CCA *p* = 0.98), **c** comparison of sample types collected at different times (*n = *20) (CCA *p* = 0.99).

Analysis of the entire study group using PCA (Fig. 5a) demonstrated overlapping clusters and canonical correspondence analysis (CCA) a p-value of 1, demonstrating no statistically significant difference in the microbial community composition between the faeces and rectal swab sample groups overall. PCA and CCA did not demonstrate any significant difference between the faeces and rectal swabs for the group of samples collected at the same time nor the group collected at different times (*p* = 0.98, *p* = 0.99) (Fig. 5b & c).

**Fig. 6:**
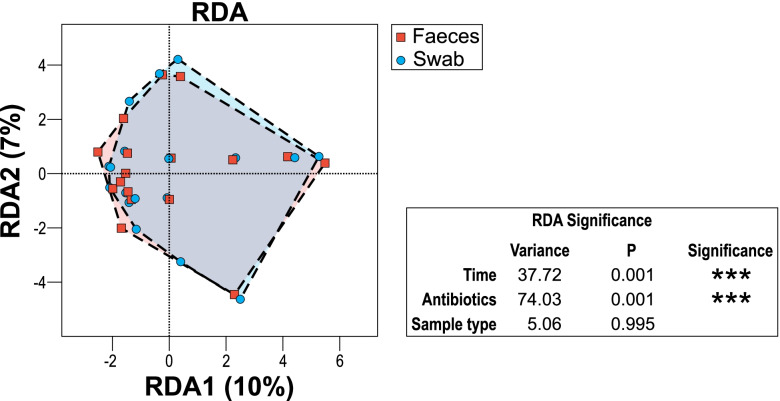
Multivariate redundancy analysis (RDA+) of OTU composition of faeces (squares) compared to swabs (circles), including a summary of the RDA significance analysis for sample type (*p *= 0.995), antibiotics (*p* = 0.001) and collection time differences (*p* = 0.001).

Multivariate RDA demonstrated significant difference between the group of samples (faeces and rectal swabs combined) collected at the same time compared to the group collected at different times (p = 0.001); and samples categorised as collected during antibiotics, after antibiotics or not on antibiotics (*p* = 0.001); but not based on sample type (*p* = 0.995) (Fig. 6). To confirm the observed relationships and to account for unknown data distributions with the selected variables, permutational manova (adonis) with Bray-Curtis distance metric was used and confirmed the finding, with time *p*-value < 0.05, antibiotics *p* < 0.05 but sample type *p* = 1.

Antimicrobial resistance (AMR) gene detection was undertaken on assembled sequences. Swabs compared to faeces had no significant difference in total contig length (*p* = 0.60) with the average total length of all contigs for faeces 200,417,113 bp (range 4,475,322 - 421,857,048), compared to swabs 187,576,694 bp (range 3,498,559- 328,988,833 bp). There were a total of 2,765,963 and 2,783,171 contigs for faeces and swabs respectively, with more than half the contigs >500 bp for both sample types (faeces compared to swabs *p* = 0.90), and average N50 for faeces 7,713 and swabs 5,392 (*p* = 0.05). Thirteen classes of AMR were detected, comprising 67 different AMR gene types, 60 in faeces and 57 in swabs. Overall, 14 (5.51%) more genes were detected in the faeces than the swabs, with 13 genes only detected in faeces and not in swabs and 19 in swabs but not in faeces (Table 5, Supplementary Table 2 and 3). The top three highest AMR gene detections were *ant*(6)-Ia_3, *Inu*(C)_1, and *tet*(Q)_1 (Supplementary Table 2 and 3). Tetracycline, beta-lactam, MLS (Macrolide-Lincosamide-Streptogramin B) and aminoglycoside resistance gene detections were the highest, noting these classes had the highest number of different genes per class detected. Of these, aminoglycoside and beta-lactam resistance genes had the highest percentage difference between faeces and swabs. This remained true for the group that did not receive antibiotics (data not shown). In view of the study objectives, the discrepancies of most interest were amongst the beta-lactamase genes, in particular any extended spectrum beta-lactamase (ESBL) or carbapenemase resistance genes.

**Table 5:** Total antimicrobial resistance gene detections by class and sample type

Class	Faeces	Swabs	Difference (%)
Glycopeptide	1	1	0(0)
Aminoglycoside	44	31	13(29.55)
Beta-lactam	45	34	11(24.44)
Phenicol	11	12	-1(9.09)
PhLOPS_A_ – Phenicols, Lincosamides, Oxazolidinones, Pleuromutilins and Streptogramin A	2	2	0(0)
Trimethoprim	3	0	3(100)
MLS – Macrolide,Lincosamide,Streptogramin B	54	57	-3(5.56)
Fosfomycin	7	7	0(0)
MDR	3	2	1(33.33)
Nitroimidazole	0	1	-1(100)
Multidrug efflux phenicol/quinolone	8	9	-1(12.50)
Sulphonamide	4	3	1(25.00)
Tetracycline	72	81	-9(12.50)
Total	254	240	14 (5.51)

Ten class A beta-lactamase gene types were detected (*bla*_ACI-1_, *bla*_SHV-187_, *bla*_SHV-32_, *bla*_TEM-116_, *bla*_TEM-1B_, *cep*A, *cfx*A3, *cfx*A4, *cfx*A5, *cfx*A6) (Supplementary Table 2). More ESBL or broad-spectrum beta-lactamase genes were detected in faeces than in swabs. A *bla*_TEM116_ gene was detected in both sample types, but 6 more *cep*A gene types were detected from faeces than swabs, however 1 swab had *cep*A_6 detected when not present in faeces. This detection was on the swab from the second paired collection, collected 7 days after the first paired collection and approximately 4 days on 6-hourly piperacillin-tazobactam. The *cep*A_6 was detected on the faeces and *cep*A_1 on both the faeces and swab for the first paired collection. Of the total of 34 *cfx*A gene detections for *cfx*A3, *cfx*A4, *cfx*A5, and *cfx*A6, there was one extra detection of *cfx*A3, *cfx*A5, and *cfx*A6 each in faeces compared to swabs. However, *cfx*A4 was detected on a swab and not the faeces, but the faeces had *cfx*A3 detected which was not present on the swab; both had 100% gene coverage.

The only class B carbapenemase gene detected in any sample type was *cfi*A13_1 which was detected on both the faeces and swab from a patient on meropenem.

Of the class C beta-lactamase genes detected, *bla*_ACC-1b_, *bla*_ACT-15_, *bla*_ACT-2_, *bla*_ACT-7_, *bla*_CMY-74_, *bla*_DHA-17_, and *bla*_PAO_, two genes were detected on faeces and not swabs (*bla*_CMY-74_ and *bla*_PAO_) and two on swabs and not faeces (*bla*_ACT-15_, *bla*_ACT-2_).

Three *bla*_OXA396_ genes were detected in faeces samples and 1 from a rectal swab. One patient had faeces with *bla*_OXA396_ detected and rectal swab not detected (pair 11), but on a later pair of samples (pair 12) both had the gene detected. This patient did not receive antibiotics. There were notable discrepancies between the number of reads for the two sample types between both pairs, with less than half the reads unmapped to the human genome (remaining reads after filtering aligned reads to the human genome) in the swab compared to the faeces for the first pair, and higher reads in the swab compared to the faeces in the second pair. These differences were also reflected in the assembled sequences. The *bla*_OXA396_ was detected on contigs identified as sequences from *Pseudomonas aeruginosa*.

Despite the variation of detections, there was no significant difference between faeces and swab groups for gene or class level detections (*p* = 0.36, *p* = 0.50), respectively. There was also no significant difference of antimicrobial resistance gene abundance determined using ALDEx2 and the Wilcoxon Rank Sum test, with the lowest p-value identified for *tet*(O)_1, *p* = 0.35. However, using a t-Test five pairs with in-pair significant differences were identified. Four had p-values between 0.02-0.04, with higher resistance gene detections for faeces in two, and in the swabs for the other two. Only one of these patients had not received antibiotics. The fifth pair had an in-pair *p*-value = 0.001, with 18 genes detected in faeces not in swabs, 3 genes in the swab not the faeces, and no genes in both sample types. The contigs with the AMR genes detected in the faeces identified as sequences from the following taxonomic orders Clostridiales (*erm*(B)_6, *ant*(6)-Ia_3), Bacteroidales (*cep*A_6), Enterobacterales (*aph*(3”)-Ib_5, *aph*(6)-Id_1, *bla*_TEM-1B_1_, *mph*(A)_2, *mdf*(A)_1, *sul*1_5, *aad*A5_1, *dfr*A17_1), Lactobacillales (*lnu*(C)_1), and one contig identified to multiple orders within the phylum Firmicutes (*aph*(3')-III_1, *ant*(6)-Ia_1), and four contigs couldn’t be reliably identified (*erm*(X)_1, *tet*(O)_1, *tet*(W)_3, *tet*(32)_2). Of the contigs with AMR genes detected only from the swab, one was identified as sequence from the order Bacteroidales (*tet*(Q)_1), and the other two contigs with the AMR genes *tet*(W)_4, *tet*(40)_1, couldn’t be reliably identified . There was no significant difference in the abundance of these taxonomic orders and phyla between the faeces and swab for this pair (*p* = 0.42), although this pair had the notable in-pair absolute difference for Clostridiales (1.77). This patient was on approximately 4 days of piperacillin-tazobactam when the samples were collected. Fewer reads remained after human reads were filtered out by mapping to the human genome for the swab (9,020,817) compared to the faeces (60,323,399) (Supplementary Table 1).

All five pairs had a higher inverse correlation between numbers of antimicrobial resistance genes detected with number of human reads in sample pairs. The correlation for reads unmapped to the human genome and number of AMR genes for these five pairs was *p* = 0.88 (*R*^2^=0.77), however in the overall cohort was *p* = 0.62 (*R*^2^=0.39). Interestingly, the two pairs with higher number of resistance genes in the swabs compared to the faeces had higher numbers of human sequence reads in the faeces than the swabs (Pair 7 and 14, Supplementary Table 1). However, these relationships between human reads and AMR resistance genes were not absolute, as some samples did not demonstrate a significant difference in AMR gene detections despite substantial differences in human reads. For example, sample pair 6 had the same 7 AMR genes detected in both faeces and swabs, with only 5% reads mapping to the human genome for the faeces and 47% for the swab, and reads remaining after human reads were filtered out by mapping to the human genome, 54,332,057 for the faeces and only 6,415,656 for the swab (Supplementary Table 1). Finally, the only OTUs with significant abundance differences based on the Wilcoxon-rank test between the swabs and faeces for these 5 pairs of collections were for *Finegoldia magna* (*p* = 0.01) and *Lawsonella clevelandensis* (*p* = 0.02). In addition, no significant difference for the overall diversity was demonstrated between the faeces and swabs (ANOVA *p* = 0.57).

## Discussion

Colonizing organisms can become lethal pathogens in ICU patients [11]. The use of techniques like WGSM that offer the ability to monitor the microbiome during ICU admission can enhance understanding of antibiotic effect on the gut microbiome as well as assist with antibiotic choice [11].

Several studies have used WGSM to examine changes in the gut microbiome due to antibiotics, including in ICU patients [2, 13]. One of the challenges for the study was regular collection of faeces samples in critically ill patients. In this paper we demonstrated the utility of rectal swabs as a surrogate to faeces if faeces collection is not possible. Although special commercial rectal swabs specifically for gut microbiome testing may be available, we investigated the use of the existing and readily available swabs in our hospital for gut microbiome testing. This would enable streamlined collection and purchasing within the existing health system, and reduce any additional expenditure, as the hospital already purchase in bulk and keep stock of standard dry cotton swabs for pathology collections. Patients are routinely screened for MDR bacteria such as carbapenemase producing Enterobacterales (CPE) in hospitals in Australia [14]. This creates an opportunity to collect a rectal swab for microbiome assessment at the same time. In this study, the rectal swabs were collected during the routine screening collections for multi-resistant organisms (MROs) from the ICU patients, at different intervals and opportunistically when patients passed faeces.

Little difference was noted between the faeces and rectal swabs in terms of organism composition. Although DNA concentration was lower in the swabs, there was no good correlation between DNA concentration and sequencing outcomes. Total number of reads and sequencing reads unmapped to the human genome were higher in faeces than rectal swabs. Unsurprisingly more human DNA was present in the swabs than the faeces, which reflects the contact with human skin and mucosa during swab collection. This is also reflected in the noted statistically significant difference in unclassified Actinobacteria, between the two groups, identified on the Wilcoxon-rank test, although when adjusted for multiple comparisons statistically significant differences were no longer observed. The phylum Actinobacteria is known for abundance on skin and in our analysis contained many common skin organisms such as *Cutibacterium, Micrococcus* and *Corynebacterium* [15]. Fair *et al* [9] reported notable differences in the abundance of Actinobacteria between rectal swabs and stool samples, cautioning against interpretation of findings from rectal swabs. However, there was no statistical significant difference for any phyla, including Actinobacteria, between swabs and faeces in our study.

No statistically significant difference was observed in overall diversity between the faeces and rectal swabs. This was consistent with findings described by Reyman *et al* [7]. In addition to the difference for unclassified Actinobacteria, the Wilcoxon-rank test indicated a statistically significant difference in abundance for a selection of anaerobes only, with the higher abundance in swabs likely representing some skin and/or vaginal organisms. This could be a reflection of the site of collection; and although some interesting differences were observed for the key anaerobic taxonomic orders, in particular for one patient from which the swab was collected just over 24 hours after the faeces, there was no statistically significant difference noted for the key anaerobic taxonomic orders selected, namely Bifidobacteriales, Bacteroidales and Clostridiales, overall. There was no statistically significant difference in abundance of *C. difficile.* A low overall abundance of this organism was noted. Testing for the presence of toxigenic *C. difficile* post antibiotics could be of value to assure any *C. difficile* findings correlate with antibiotic effect, given the demonstrated knowledge of *C. difficile* infection following antibiotics [16]. There was also no statistically significant difference in any of the other key organisms investigated such as Enterobacterales, which is of great interest due to the potential for creating a reservoir of multi-resistant Gram-negatives in the bowel [10]. Overall sample type was not demonstrated to predict variation in microbial communities; however antibiotics and difference between time of faeces and rectal swab collection were both considered significant influences.

There was variability in the antibiotic resistance genes detected from faeces and rectal swabs. Overall, more resistance genes were detected from faeces samples. Several discrepancies in detections were noticed, in particular in one patient an oxacillinase gene was not detected on the swab but detected in the faeces and later detected in both sample types. Five pairs had significant differences in gene detections, although cautious interpretation of low numbers has to be taken into account. The differences were pronounced in a paired collection where much higher numbers of human reads were generated from the swab, and most notably the faeces had several aminoglycoside genes, and a *bla*_TEM1B_, which were not detected from the swab. There was an inverse correlation between higher human reads and AMR genes in samples, however this was not an absolute finding, as some samples did not demonstrate this difference. Future studies may need to be undertaken to understand the drivers for the observed discrepancies in more details, especially given the abundance differences between the swabs and faeces for the pairs with significant AMR gene differences had no additional OTUs identified as significantly different between the faeces and swabs compared to the overall cohort. Regardless, these discrepancies indicate cautious use of rectal swabs as surrogate for faeces collections for resistome monitoring, especially when a high proportion of reads map to the human genome. Given some of these notably different samples were in fact swabs with higher AMR genes and faeces with the higher human reads, the importance of ensuring adequate read output quantity is highlighted. Although the discrepancies between swabs and faeces for AMR detection may reflect limit of detection of WGSM processes, it also raises the question about the possible use of faeces for routine resistance gene screening instead of the current rectal swab screening [14], given the higher resistance gene detection possibility in faeces. The collection of faeces for MDR organism detection will also enable microbiome testing for monitoring of organism composition and pathogen selection from antimicrobials [2].

Limitations for our study include the defined patient cohort examined and the small sample size. However, this study is described to assess the utility of rectal swabs collected with the specific aims addressing effects of antibiotics on the gut microbiome. The small number of samples available for analysis in this study reflects the challenge which the rectal swabs are able to assist in addressing, namely lack of faecal sample collection on regular intervals during intensive care admission [4]. Another limitation is that several antibiotic resistance genes were present in a low number of samples, making the assessment of true discrepancies difficult. Finally antibiotic resistance gene detection was qualitative not quantitative, therefore the lack of detection due to possible low level gene presence was also not determined.

## Conclusion

We have demonstrated that rectal swabs collection, with standard dry cotton swabs, could be used for gut microbiome assessment observation of diversity and key organisms selected in our study to monitor the effects of antibiotics on the gut microbiome, if faeces are not able to be collected. However, when assessing changes between samples collected at different time points in a single individual, caution has to be applied to interpretation of low abundance organisms, and analysis should be aimed at a taxonomic level or specific marker genes such as toxin genes used to assist in interpretation of changes. Antibiotic resistance gene detection comparison between sample types have to be undertaken with caution and quantitative measure of gene detections may need to be considered to observe antimicrobial impact. Demonstrating the use of existing sampling techniques in hospitals for gut microbiome testing will hopefully grease the path for introduction of gut microbiome testing in practice to assist with antibiotic choice.

## Materials and methods

The aim of the study was to compare the utility of rectal swabs as a surrogate for faeces collection from patients in ICU to monitor the effects of antibiotics on the gut microbiome during admission. The two collection techniques were compared in terms of microbial diversity, and the abundance of key organisms of interest due to association with antibiotic affect, namely, Enterobacterales, *C. difficile*, *Pseudomonas* spp., *Acinetobacter* spp., *Candida* spp., and organisms considered to be anaerobic (Bacteroidales, Bifidobacteriales, Clostridiales and Fusobacteriales); and resistance genes in the gut metagenome [10, 11].

### Sample collection

A patient cohort was selected that are usually not on antibiotics when admitted, were likely to stay in ICU for >3 days and often require antibiotic treatment during their admission due to infections. Faeces and rectal swabs were collected from patients 18 years or over, admitted to the ICU in just over a 1 year period, with trauma, non-traumatic sub-arachnoid haemorrhage (SAH), or burns at the Royal Brisbane and Women’s Hospital (RBWH), Queensland, Australia. Approval from the RBWH Human Research Ethics Committee was obtained (HREC/16/QRBW/463).

Patients on antibiotics at the time of admission and who received broad spectrum antibiotics such as piperacillin-tazobactam or meropenem in the last 30 days prior to admission were excluded. Patients not expected to survive more than 4 days, or receiving any form of bowel disinfection treatment, or pregnant or breast-feeding were also excluded from the study.

Rectal swabs used were standard dry cotton swabs with no transport medium. All samples were sent to the laboratory, where they were frozen at -20 ֯C and then transferred on dry ice to the -80 ֯C freezer as soon as possible. Samples were kept frozen until processed and were thawed immediately prior to DNA extraction.

### DNA extraction

DNA extraction was performed based on the method by Yu and Morrison [17]. Briefly, each sample (swab or 0.15 gram faeces) was mixed with 600 μl lysis buffer and sterile zirconia beads (0.1 mm – 1mm). After bead-beating, using the Precellys® 24 Homogenizer (Bertin, France), the samples were incubated (70°C for 15 minutes, with gentle shaking by hand every 5 minutes), centrifuged at 4°C for 5 minutes 13,200 rpm, and the supernatant further treated using 30 μL Proteinase K by vortexing and then incubating at 56°C for 20 minutes. DNA extraction was performed using the Maxwell® 16 MDx (Promega Corporation, USA) system as per the manufacturer’s instructions. Paramagnetic particles were removed by centrifugation at 10,000 g for 2 minutes, and then the supernatant was removed and incubated at 37°C for 15 minutes with 2μL RNase (10 mg/ml). For rectal swabs the method was modified by removing the lysis buffer after the bead-beating, repeating the lysis buffer suspension bead-beating step and then combining the lysis buffer from both steps into one tube for the remaining protocol. A blank control was included with each extraction run and subsequently sequenced for quality control and monitoring of possible contamination.

### Whole genome sequencing metagenomics

For library preparation, the DNA concentration for each sample was determined using the Quant-iT^TM^ dsDNA High-Sensitivity Assay Kit (Thermo Fisher Scientific Inc., USA), as per the manufacturer’s instructions.

The library preparation was undertaken according to the Nextera ® XT DNA Library Prep Guide (Illumina Inc., USA) as per the manufacturer’s instructions, with modification to the input DNA concentration to 0.2 ng/μL. The protocol used prepared indexed paired-end libraries from DNA, for sequencing on the Illumina NextSeq® 500 System (Illumina Inc., USA). Briefly, the extracted DNA was tagmented (i.e. fragmented and adapter sequences added) and the resulting tagmented DNA was PCR amplified (PCR primers: 5'-AATGATACGGCGACCACCGA and 5'-CAAGCAGAAGACGGCATACGA, Oligonucleotide sequences © 2016 Illumina, Inc.) and indices added. The DNA was then purified and size selected (300-400 bp) to remove short library fragments. The libraries were normalized to ensure equal representation and were then pooled by combining equal volumes of the prepared libraries, before sequencing (Nextera ® XT DNA Library Prep Reference Guide, Illumina Inc, USA).

Prepared libraries were denatured and diluted based on the manufacturer’s instructions (NextSeq® Denature and Dilute Libraries Guide, Illumina Inc., USA), with modification of loading concentration to 1.2 pM, as per the laboratory protocol at Queensland Health Forensic and Scientific Services, based on previous verification. Whole genome sequencing was undertaken on the NextSeq® 500 System (Illumina Inc., USA) as per the manufacturer’s instructions. Briefly, a reagent cartridge and flow cell was prepared and the software prompts followed. Cluster generation and sequencing was then performed by the system (NextSeq® 500 System Guide, Illumina Inc., USA). Paired end 2 x 150 bp sequences were generated.

### Analytics

Basecalling was performed using bcl2fastq v2.17.1.14 (Illumina, USA). For trimming and demultiplexing, and quality of assessment bcl2fastq and Trimmomatic v0.36 [18] were used. Trimmomatic removes the adapters as well as low quality bases (<Q30) from the beginning and ends of the reads. Average length after trimming was 127 bp. Using CLC Genomics Workbench version 8.0.2 (Qiagen, Germany), all reads were mapped against the human reference genome (NCBI Accession number GCA_000001405.1). Unmapped reads were uploaded to and bioinformatically analysed using One Codex (One Codex, USA) with the 2019 database. One Codex uses a *k*-mer based analysis against a curated in-house database. Sequences are identified by exact alignment using *k*-mers (*k* = 31 bp). Overlapping *k*-mers in a read are matched to the most specific organism possible, classification is to the lowest common ancestor taxon, and *k-*mers are aggregated across a given read to ensure specific and consistent taxonomic assignment [19, 20]. The output contains the number of sequences assigned for each taxonomic unit for every sample in .biom file format. Output for taxonomies rolled up into the different taxonomic level is available in comma separated file format.

To observe changes during admission and to account for any pre-admission gut microbiome alteration in-pair comparison to the baseline sample collected for each patient was implemented. To assess the utility of rectal swabs when faeces samples were not available, especially as baseline sample, pairs of faeces and rectal swab samples collected about 24 hours apart (one pair was 24.5 hours apart, all other pairs were <24 hours apart) were compared in terms of the aims and objectives set out. Statistical significance of differences between samples was assessed using Calypso v.8.84 (Zakrzewski et al., 2016), and the default parameters. The OTU .biom output from One Codex was used for overall statistical analysis and the rolled up taxonomic level data for the specific interrogation of the key organism groups of interest. Calypso is a web-application specifically designed to compare and interpret taxonomic information from microbial metagenomic sequence compositional data. The software allows the user to select from several statistical analysis methods and graphical displays which can be applied to the uploaded data. For this study the output from reads assigned to taxons from One Codex (One Codex, USA) and meta-information prepared by the authors for each sample was uploaded into Calypso [21]. Uploaded read data was normalised using the default parameters which is set to include the top 3000 taxa, and exclude samples with less than 1000 sequence reads and taxa with less than 0.01 percent relative abundance across all samples. Total sum normalisation and square root transformation (Hellinger transformation) was used to render the compositional data suitable for standard statistical analysis [22]. The software was used to perform abundance determination and commonly used statistical testing and visualisation of taxonomic information, including diversity estimates using multiple metrics, regression, analysis of variance (ANOVA), Wilcoxon signed-rank testing, principal component analysis (PCA), canonical correspondence analysis (CCA), redundancy analysis (RDA) and multivariate analysis [21]. In addition, Calypso was used to determine false discovery rate (FDR) and Bonferroni correction, which were applied to counter potential statistical type I errors due to multiple comparisons. Calypso [21] was also used for linear distribution (multivariate RDA) and non-parametric (permutational manova) data analysis to assess the association between microbial community composition and multiple explanatory variables [23]. Additional statistical analysis of reads and in-pair abundance comparisons was performed using Microsoft 365 Excel ® with the Data Analysis Toolpak Add-in. A *p*-value of <0.05 was considered statistically significant. Values >0.01 were rounded to two decimal places and lower values to the first non-zero decimal number. Figure 1 was produced using Excel and Figs. 2-6 using Calypso with graphical modification for improved visualisation.

To determine differences in acquired antibiotic resistance genes, assembly was performed using MegaHit v1.2.9 [24] and resistance genes detected using Abricate v1.0.1 [25] with the default settings and ResFinder database (2020-Dec-13) available at the time [26]. Quality of assemblies were assessed using QUAST v4.6.3 [27]. The number, length and N50 for all contigs were determined. Contigs were not excluded based on length and all contigs were utilised for gene detection, however quality of gene detection was further managed through Abricate [25]. Abricate is a bioinformatic tool for the mass screening of contigs for acquired antimicrobial resistance genes by using DNA sequence matching against the selected antimicrobial resistance gene database. Results can be combined into a matrix with the presence of genes indicated by the percentage coverage of the gene. The default settings were used with a cut-off for gene coverage a minimum of 80% and the same for percentage identity. Resistance gene detection was based on the presence or absence of genes, which included counting two detections of the same gene in a sample as a single detection and two different genes for the same resistance gene type as a single gene type in a sample, was counted as single gene type detection. For example *tet*(32)_1 and *tet*(32)_2 were counted as two genes but rolled up into a single gene type detection, *tet*(32). Gene class allocations were based on information from the Resfinder database [26, 28], Abricate v1.0.1, and NCBI MicroBIGG-E [29]. Statistical analysis for contigs and resistance gene differences were calculated using Microsoft 365 Excel ® with the Data Analysis Toolpak Add-in. Values were compared and tested using t-Test and p-value of < 0.05 was considered statistically significant. Additional resistance gene statistical analysis was performed using ResistoXplorer [30]. In brief, the summary gene detection data from Abricate was utilised together with metadata and a resistance gene key for uploading to ResistoXplorer. This web-based tool performs a wide variety of statistical analysis of resistome data. ALDEx2, an ANOVA-like differential expression tool, was utilised with the Wilcoxon test to determine ranking of resistance gene abundance with a Monte Carlo number set at the default of 64 samples. ALDEx2 estimates variation within samples using Monte-Carlo instances and using centered log-ratio transformation [30, 31]. Further identification of contigs with AMR genes was performed using the NCBI Basic Local Alignment Tool (BLAST) [32], using the default parameters and standard nucleotide collection (nr/nt). For each contig the taxonomic order for the organism/s with the highest percentage identification (cut-off > 99%) in combination with sequence cover (cut-off >95%) was used. Contigs with multiple identifications with the same scores had to belong to a single taxonomic order or phylum for inclusion in the results.

## Supplementary Information

**Additional file 1:** **Supplementary Table 1.** DNA concentration, read numbers, diversity, and key organism abundance for each sample pair. **Supplementary Table 2.** Antimicrobial resistance genes found in collectively in all faeces and swab samples. **Supplementary Table 3.** Gene detection discrepancies. Genes with additional detections for each sample type.

## Data Availability

The data that support the findings are not publicly available as it contains human data and information that could compromise research participant privacy/consent and ethical approval. Requests for data can be sent to the corresponding author (SS), and will be reviewed by the relevant Human Research Ethics Committee and the University of Queensland to verify if the request is subject to any intellectual property or confidentiality obligations. Patient-related data not included in the paper might be subject to patient confidentiality. Any data and materials that can be shared will likely be released via a Material Transfer Agreement. All software used is publicly (open source or commercially) available software.

## Abbreviations

MDR: Multi-drug resistant
ESBL: Extended spectrum β-lactamase
WGSM: Whole genome sequencing metagenomics
SAH: Sub-arachnoid haemorrhage
ICU: Intensive Care Unit
RBWH: Royal Brisbane and Women’s Hospital
HREC: Human Research Ethics Committee
ANOVA: Analysis of variance
PCA: Principal component analysis
CCA: Canonical correspondence analysis
RDA: Redundancy analysis
FDR: False discovery rate
MANOVA: Multivariate analysis of variance
OTUs: Operational Taxonomic Units
AMR: Antimicrobial resistance
CPE: Carbapenemase producing Enterobacterales
bp: Base-pairs
BLAST: Basic Local Alignment Tool
MLS: Macrolide, Lincosamide, Streptogramin B
PhLOPS_A_ (33): Phenicols, Lincosamides, Oxazolidinones, Pleuromutilins and Streptogramin A
MROs: Multi-resistant organisms
